# Neuropeptide Substance P Enhances Skin Wound Healing In Vitro and In Vivo under Hypoxia

**DOI:** 10.3390/biomedicines9020222

**Published:** 2021-02-22

**Authors:** Suneel Kumar, Yuying Tan, Francois Berthiaume

**Affiliations:** Department of Biomedical Engineering, Rutgers, State University of New Jersey, 599 Taylor Road, Piscataway, NJ 08854, USA; sk1350@soe.rutgers.edu (S.K.); minervatan27@gmail.com (Y.T.)

**Keywords:** neuropeptides, substance P, normoxia, hypoxia, starvation, proliferation and migration, wound healing, in vivo and in vitro, histology

## Abstract

Pressure ulcers (PUs) or sores are a secondary complication of diabetic neuropathy and traumatic spinal cord injury (SCI). PUs tend to occur in soft tissues located around bony prominences and may heal slowly or not at all. A common mechanism underlying impaired healing of PUs may be dysfunction of the local neurovascular system including deficiency of essential neuropeptides, such as substance P (SP). Previous studies indicate that disturbance in cutaneous sensory innervation leads to a defect in all stages of wound healing, as is the case after SCI. It is hypothesized that nerve fibers enhance wound healing by promoting initial inflammation via the releasing of neuropeptides such as SP. Therefore, we investigated whether exogenous SP improves skin wound healing using in vitro and in vivo models. For in vitro studies, the effects of SP on keratinocyte proliferation and wound closure after a scratch injury were studied under normoxia (pO_2_ ~21%) or hypoxia (pO_2_ ~1%) and in presence of normal serum (10% *v*/*v*) or low serum (1% *v*/*v*) concentrations. Hypoxia and low serum both significantly slowed cell proliferation and wound closure. Under combined low serum and hypoxia, used to mimic the nutrient- and oxygen-poor environment of chronic wounds, SP (10^−7^ M) significantly enhanced cell proliferation and wound closure rate. For in vivo studies, two full-thickness excisional wounds were created with a 5 mm biopsy punch on the dorsum on either side of the midline of 15-week-old C57BL/6J male and female mice. Immediately, wounds were treated topically with one dose of 0.5 μg SP or PBS vehicle. The data suggest a beneficial role in wound closure and reepithelization, and thus enhanced wound healing, in male and female mice. Taken together, exogenously applied neuropeptide SP enhanced wound healing via cell proliferation and migration in vitro and in vivo. Thus, exogenous SP may be a useful strategy to explore further for treating PUs in SCI and diabetic patients.

## 1. Introduction

Normal wounds self-heal following a typical wound repair process, which involves four temporally and spatially overlapping phases: coagulation, inflammation, proliferation, and remodeling [1]. A prolonged inflammatory phase leads to chronic wounds such as pressure wounds or sores or ulcers (PUs), which do not heal in a predictable amount of time [2]. PUs are a common but serious complication of spinal cord injury (SCI) and neuropathy due to diabetes and age, and tend to occur in soft tissues (i.e., skin and underlying fat and muscle) located around bony prominences where body weight is concentrated while the patient is sitting or lying [3,4]. A multifactorial process contributes to PU formation, including extrinsic causes such as local pressure, temperature, and moisture, as well as intrinsic factors such as the patient’s mechanical properties of bone, muscle, and soft tissue surrounding the wound [5]. PUs often heal slowly and have a high risk of life-threatening complications such as infection and pneumonia [6]. Three million people are suffering from PUs in the US alone, and 60,000 die from PU complications annually. The annual cost of PU care is $11 billion [7]. Therefore, PUs are a prevalent and costly health problem nationwide. Current treatments include local wound care, negative pressure wound therapy, electrical stimulation, and surgery, which are either too invasive or have limited therapeutic outcomes [7]. Thus, better treatment for PUs is an urgent need.

Sensory neurons and their derived neuropeptides have been reported to play an important role in the wound healing process, especially for the inflammation and proliferation phases. Disturbance in the cutaneous nerve fibers delayed all phases of wound healing [8,9,10]. Reduction of neuropeptides, such as substance P (SP), may induce a chronic wound [11]. SP is an important neuropeptide for wound healing and immune activities [12]. The positive therapeutic effect of SP on wound healing in diabetic models has been widely studied [11,13,14]. It was reported that SP levels decreased in diabetic wounds and that topical application of exogenous SP enhanced wound healing significantly in a diabetic rat wound model [11]. Several studies show that SP can also improve the healing of other types of wounds, including burns and photodamaged corneas [15,16,17,18,19]. Faden’s study reported that local SP content at the SCI site decreased in a rat model, and Lenoard et al. showed that SP decreased in the dorsal horn of human SCI tissue [20,21]. Based on these clues, it is plausible that exogenous SP could also improve the healing of PUs in SCI. Aging is also related to slow or defective wound healing due to less inflammatory signal via cutaneous nerve fibers, as confirmed by rodent and human studies [3,22].

Therefore, we hypothesized that exogenous SP application can improve the healing process of chronic wounds. We therefore examined the effect of SP in vitro under hypoxic and limited serum conditions, and in vivo in 15-week-old mice, to simulate the chronic wound condition of slow healing. We used an in vitro scratch assay to optimize the concentration of SP and found that 10^−7^ M SP could significantly improve wound closure under hypoxia and low serum concentration. A single application of exogenous SP accelerated the closure of full-thickness wounds in 15-week-old male and female mice.

## 2. Experimental Section

### 2.1. Cell Culture Chemicals and Reagents

HaCaT cells (P37-39, immortalized keratinocytes), Dulbecco’s modified Eagle’s medium (DMEM containing 4.5 g/L glucose), and fetal bovine serum (FBS) were from Life Technologies (Carlsbad, CA, USA). Additionally, 1% *v*/*v* penicillin–streptomycin (PenStrep) was from Sigma Aldrich and AlamarBlue™ cell viability reagent was from Invitrogen. Fluorescence readings were taken using a microplate reader (DTX 880 Multimode Detector, Beckman Coulter, CA, USA). Hypoxic cultures were carried out in a hypoxia incubator (Galaxy^®^ 14 S CO_2_ Incubators, Eppendorf, Framingham, MA, USA).

### 2.2. Cell Culture and Maintenance

HaCaT cells were cultured in T75 flasks in DMEM supplemented with 10% FBS and 1% PenStrep. The media were changed every 72 h until cells were sufficiently confluent to the plate for proliferation or scratch wound assays. For these experiments, cells were incubated under normoxia (N; 21% O_2_, 5% CO_2_, balance N_2_) or hypoxia (H; 1% O_2_, 5% CO_2_, balance N_2_), and 100% humidity at 37 °C for 24–72 h.

### 2.3. Cell Proliferation/Metabolic Activity

**Figure 1:**
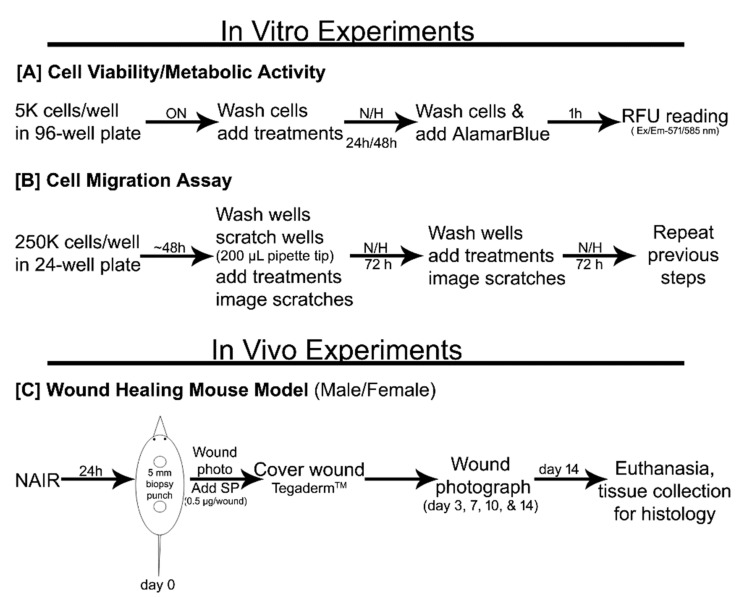
Schematic representation of the whole study design. (**A**) In vitro cell proliferation study after 24 h and 48 h incubation. (**B**) In vitro wound healing study for 6 days using a scratch assay. (**C**) Full-thickness skin wound healing (biopsy punch) study in male and female mice. ON: overnight; N: normoxia; H: hypoxia, RFU: relative fluorescence units, SP: substance P.

The experimental timeline is summarized in Figure 1A. HaCaT cells (5000/well) were plated in a 96-well plate in DMEM with 10% FBS overnight (12–16 h). The next day, cells were washed with PBS twice and media were replaced with DMEM with no FBS supplementation. Treatments were added, namely, 10^−7^ M SP (SP group) or no stimulus (control group). Plates were then incubated for 24–48 h under normoxia or hypoxia conditions. The total cellular metabolic activity in each well was measured at 24 h and 48 h. For this purpose, AlamarBlue (10% in DMEM) was added to the wells according to the manufacturer’s instructions, and cells were incubated for 1 h in their respective incubator. Fluorescence (Ex/Em-571/585 nm) in each well was read in a microplate reader and reported as relative fluorescence units (RFU).

### 2.4. Cell Migration (Scratch Assay)

The experimental timeline is summarized in Figure 1B. HaCaT cells were plated (250,000/well) in a 24-well plate and allowed to reach confluence (~48 h). At that point, and before any medium change, a scratch was made in each well using a sterile 200 μL pipette tip to create an “injury” in the center of the HaCaT monolayer. The wells were then washed with 1× PBS twice before introducing the experimental conditions, including 1% FBS or 10% FBS supplementation and under normoxia or hypoxia, and SP at different concentrations (0, 10^−6^, 10^−7^, 10^−8^ M). The data of SP 10^−7^ M are discussed here in detail and the other two concentrations (10^−6^ and 10^−8^ M) were used for standardization (Blais et al., 2014) along with 10^−7^ M SP (Figure S1). The wells were photographed using an Olympus CKX41 light microscope using a 10× objective to capture the initial wound area (day 0). The plates were incubated in different conditions for the next 3 days. Wells were washed with PBS (1×) and refreshed with the respective experimental conditions every 3 days until the scratch wound completely closed. Scratch area images were captured at every time point and quantified by ImageJ analysis software (NIH). The wound area in each well was normalized to its area at the initial time point and expressed as a percentage of wound closure. The equation used was: Percentage wound closure = (1 − (remaining wound area)/(initial wound area)) × 100%.

### 2.5. Skin Wound Healing Animal Study

Animal studies were performed in accordance with a protocol approved by the Institutional Animal Care and Use Committee at Rutgers University (IACUC ID: PROTO999900017 approved on 29 August 2017). Fifteen-week-old male and female mice (C57BL/6J, inbred in-house colony from Rutgers University, were used in this study.

#### 2.5.1. Skin Full-Thickness Wound Model

The experimental timeline is summarized in Figure 1C. One day before surgery, mice (male and female) were anesthetized by isoflurane (Henry Schein, Melville, NY, USA) inhalation, and the back of each mouse was shaved using a clipper and depilated using Nair^TM^ cream (Church & Dwight Co., Inc., Ewing, NJ, USA). On the day of surgery, the mice were anesthetized, and betadine scrub and 70% ethanol were applied alternately 3 times to prepare the dorsum for wounding. A 5 mm biopsy punch (Integra Life Sciences, Princeton, NJ, USA) was then used to create two circular full-thickness skin wounds on either side of a median line on the dorsum, approximately ~1 inch apart. The mice were randomly divided into two groups, namely the SP group and the control vehicle group, and immediately treated once with 0.5 μg/wound SP or plain PBS, respectively. The wounds were then covered with Tegaderm^TM^ (3M, Saint Paul, MN, USA). The wound area was photographed immediately on day 0 and then on days 3, 7, 10, and 14. The percentage of wound closure was analyzed using ImageJ software (NIH) and calculated as described above (for the in vitro scratch studies).

#### 2.5.2. Skin Wound Histology

On post-wounding day 14, mice were sacrificed and skin samples around the wound area (including the scar area) were excised. The collected tissues were processed for histology as previously described. Briefly, the tissues were fixed in 10% neutral buffered formalin (VWR; Radnor, PA, USA) for 72 h and later stored at 4 °C in 70% ethanol until processing for histology. Tissues were paraffin embedded and sectioned (5 μm thickness). The sectioned tissues were stained with hematoxylin and eosin (H&E) stain and imaged using a light microscope (2.5×). Studied histological parameters included the thicknesses of epidermis and dermis that were compared among treatment groups for male and female mice.

### 2.6. Statistical Analysis

Statistical analysis was performed using KaleidaGraph software (version 8.4) and graphs were prepared using GraphPad Prism (version 8.4.3). Differences among experimental groups were analyzed using one-way ANOVA followed by a post hoc Fisher’s least significant difference (LSD) test, or a Student’s *t*-test for comparisons limited to two groups. The results are expressed as mean ± standard error of the mean (SEM). A *p*-value < 0.05 is considered statistically significant.

## 3. Results

### 3.1. Effect of SP and Hypoxia on Cell Proliferation In Vitro

**Figure 2:**
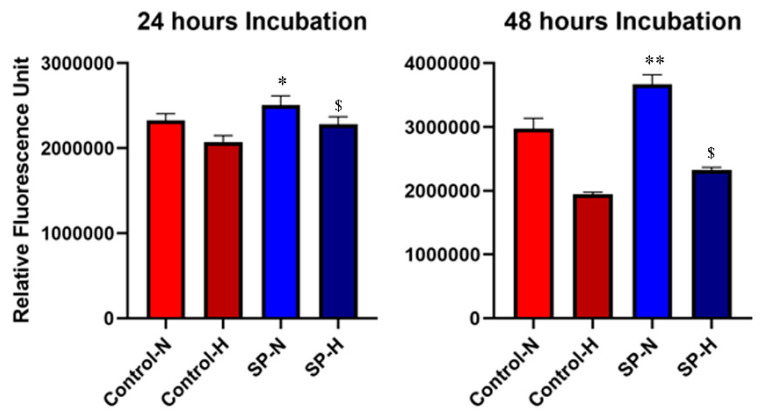
Evolution of viable cell number in normoxic (N) and hypoxic (H) conditions in absence (control) or presence of 10^−7^ M SP (SP). HaCaT cell number was estimated by the metabolic reduction of AlamarBlue per well. Cells were incubated in serum-free Dulbecco’s modified Eagle’s medium (DMEM) in the specified conditions (*n* = 6). */** indicates a comparison between control and SP groups under normoxia (N). $ indicates a comparison between control and SP groups under hypoxia (H). */** *p* < 0.05/0.01, while ^$^ *p* < 0.05.

Human HaCaT cell proliferation, measured per well, was determined over 48 h in serum-free conditions under normoxia or hypoxia, and in the presence or absence of the neuropeptide SP (10^−7^ M). Hypoxia decreased (*p* = 0.008 at 24 h and *p* = 0.0002 at 48 h) the viable cell number as compared to normoxia. Overall, SP increased proliferation both at 24 h (ANOVA; F = 11.78, *p* < 0.01) and 48 h (ANOVA; F = 45.11, *p* < 0.0001). Post hoc test analysis suggested that adding SP to the culture without serum enhanced the cell proliferation in normoxia (*p* = 0.04 at 24 h, *p* = 0.002 at 48 h) and hypoxia (*p* = 0.02 at 24 h, *p* = 0.04 at 48 h) at both time points (Figure 2). This suggests that SP at this concentration promotes cell proliferation in a serum-free medium under both normoxic and hypoxic conditions.

### 3.2. Effect of SP on Wound Closure In Vitro

**Figure 3:**
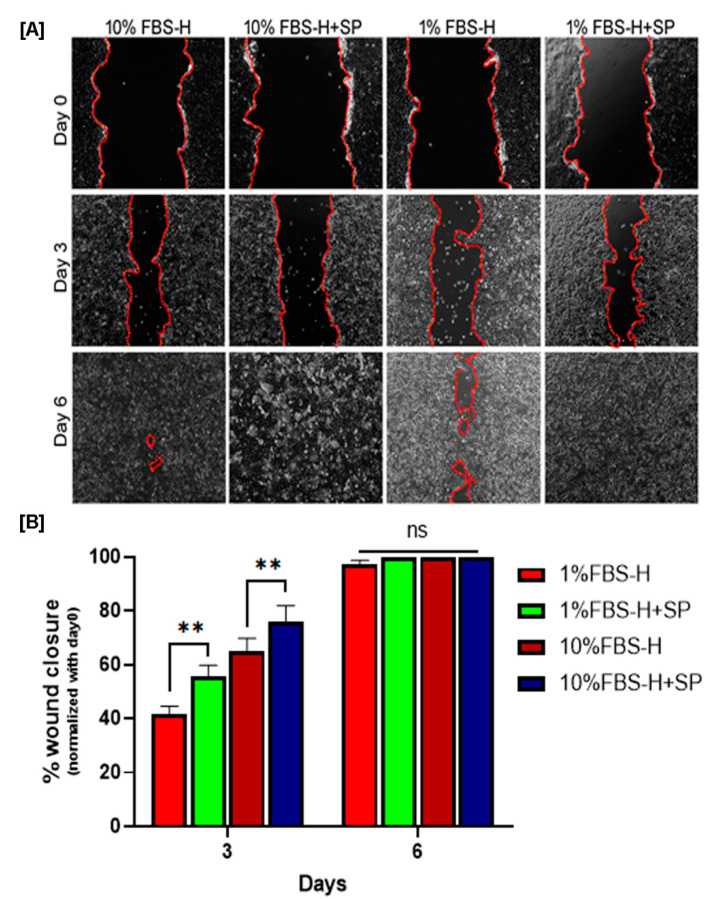
Effect of SP on wound closure in vitro under hypoxic and/or low-serum conditions. Representative images of scratch injuries in confluent HaCaT cultures (*n* = 8–11) in each group over 6 days (**A**). Quantified wound images showing the fraction (%) of wound closure (**B**). ** *p* < 0.01. FBS: fetal bovine serum.

A scratch assay was carried out to test the effect of SP in combination with hypoxia (1% vs. 21% O_2_) and low serum (1% FBS vs. 10% FBS) on the in vitro wound healing response of HaCaT cells. We observed that all scratch wounds closed by day 6 except for the 1% FBS group under hypoxia (Figure 3). There was also a significant slowing of wound closure rate in the 1% FBS group vs. the 10% FBS group throughout the study period (*p* < 0.01). Supplementation with SP (10^−7^ M) caused better wound closure at day 3 than untreated cells under conditions of hypoxia and 10% FBS (*p* < 0.01). Similarly, under conditions of hypoxia and low serum (1% FBS-H), the SP treatment group had better wound closure at day 3 than the untreated group (*p* < 0.01) (Figure 3, lower panel). Thus, SP improved wound closure in this assay under conditions that mimic chronic wounds, namely, hypoxia and low serum availability.

### 3.3. Effect of SP on Wound Closure In Vivo

**Figure 4:**
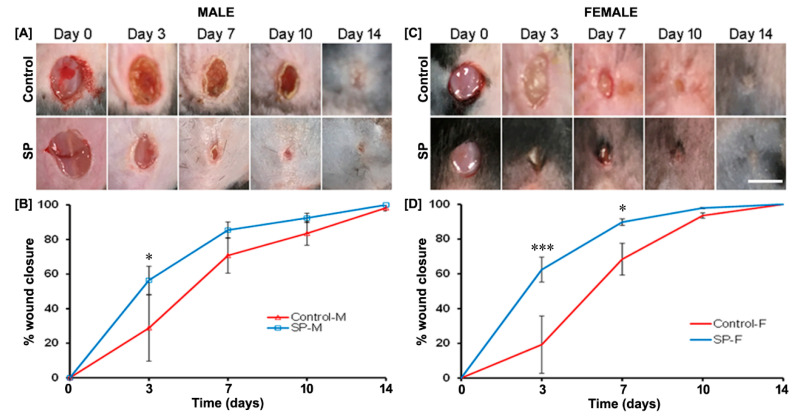
Effect of SP treatment on male and female mouse wound closure. Representative images of skin wounds in control and SP-treated male (M, *n* = 4) and female (F, *n* = 4) mice over 14 days after 5 mm biopsy full-thickness wounding (**A**,**C**, respectively). Scale bar = 5 mm. Quantified wound images showing the fraction of wound closure as a function of time (**B**,**D**). */*** indicates a comparison between control and SP-treated groups. * *p* < 0.05 while *** *p* < 0.001.

To study the effect of neuropeptide SP in vivo, a full-thickness skin wound was created and treated immediately with a single topical dose of neuropeptide SP in 15-week-old male and female mice (Figure 4A–D). There was an overall difference between male and female control mice (data not shown) over 14 days (ANOVA; F = 8.74, *p* = 0.0001) in the wound healing closure rate, although no statistically significant difference between the two groups could be determined for any specific time point. We found that SP treatment accelerated wound healing in both male (ANOVA; F = 7.6644, *p* = 0.0001) and female (ANOVA; F = 15.331, *p* = 0.0001) mice as compared to their respective vehicle control (Figure 4B,D, respectively). In male mice, the percentage of wound closure was statistically significantly higher in the SP-treated group vs. control on day 3 (*p* = 0.035) (Figure 4B). In female mice, a significant difference vs. vehicle control was seen both on day 3 (*p* = 0.0003) and day 7 (*p* = 0.045) (Figure 4B).

**Figure 5:**
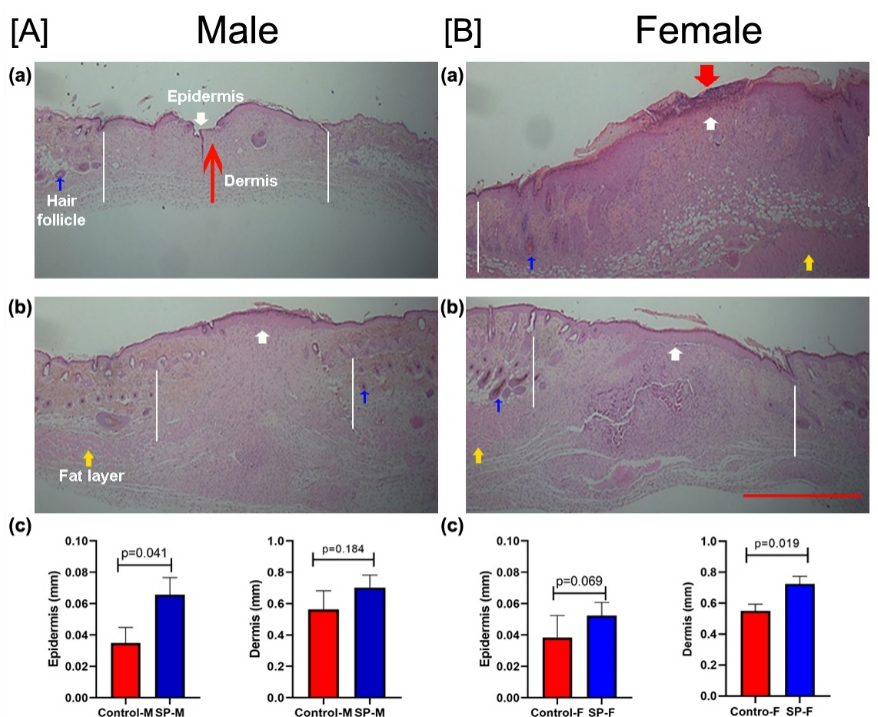
Effect of SP treatment on male (M) and female (F) mouse wound histology. Representative histology of healed wound skin (hematoxylin and eosin (H&E) stain) showing epidermis (white arrow for the reappearance of the epidermis and red thick downward arrow for missing epidermis), dermis (red thin arrow), hair follicles (blue arrow), and fat layer (yellow arrow) in the male and female untreated control (**Aa** and **Ba**, respectively) and SP-treated (**Ab** and **Bb**, respectively) mice. Scale bar = 500 µm. Quantification of the epidermal and dermal thickness of healed wounds on post-wounding day 14 in male (**Ac**) and female mice (**Bc**). White lines on each side of the image represent the wound width. Statistical significance was determined by the Student’s *t*-test.

After the end of the study period on day 14, skin wound tissues were processed for H&E staining to assess the morphology and measure the thicknesses of the epidermis and dermis of the healed wound (Figure 5). The wound linear width (which reflects scar diameter) was found to be smaller in the SP treatment groups compared with the controls for each sex. Wound width was generally larger in females. In male mice, all animals had healed, as defined by a reconstituted epidermis with no gap, and also exhibited a dermal scar (Figure 5A,B). We assessed the thickness of these layers at three sites, namely, the center of the wound and two wound edges. In the SP-treated males, epidermis (*p* < 0.05) and dermis (*p* > 0.05) were thicker than in untreated control mice (Figure 5Ac). In control female mice, one out of four mice exhibited partial reconstitution of the epidermis. Hypertrophy of the epidermis suggests that the wound was still in the remodeling phase (Figure 5Ba). On the other hand, all SP-treated female mice had fully regenerated epidermis and dermis (Figure 5Bb). In SP-treated female mice, epidermis (*p* > 0.05) and dermis (*p* < 0.05) were thicker than in untreated control mice (Figure 5Bc).

## 4. Discussion

In this study, we investigated the potential of exogenous SP to improve wound healing under unfavorable conditions, such as restricted serum and hypoxia in vitro using keratinocytes, and in full-thickness wounds using 15-week-old male and female mice in vivo. In vitro data suggested that hypoxia and low serum concentration significantly reduced the rate of cell proliferation and wound healing, whereas exogenous 10^−7^ M SP significantly enhanced cell proliferation as well as wound healing in a scratch assay with or without serum restriction in a hypoxia environment. This implies SP has a distinct function in wound healing enhancement rather than a simple replacement of serum. Topical application of SP in vivo resulted in improved healing of experimental full-thickness skin wounds in 15-week-old male and female mice. Wound histology showed that epidermis and dermis were significantly thicker in SP-treated male and female mice in comparison to non-treated mice.

Keratinocytes form a barrier against pathogens which is a primary function of the skin. Keratinocytes are primarily responsible for restoring the epidermis after any injury to the skin. The defining parameters of successful wound closure are characterized by keratinocyte proliferation, migration, and ultimately differentiation [23,24]. Impaired or slow wound healing is a common complication of traumatic SCI injury-induced immobility [25], aging [26], and other pathological conditions such as diabetes and hypertension [24]. Delayed wound healing is generally characterized by a weak and often prolonged inflammatory phase with persistent infections, thereby negatively affecting cell proliferation and migration [27]. Impaired blood flow causing limited nutrient and oxygen supply (hypoxia) is thought to be a major underlying cause in the delayed repair process [28]. We found previously that serum starvation as well as hypoxia significantly slow down the proliferation and migration of keratinocytes in vitro [29], which is comparable to this study. In the current in vitro study, we chose keratinocytes as they are the primary cells to close the wound and they also express the receptor for SP. Exogenous application of SP restored cell proliferation and cell migration.

Studies from other groups also suggested that keratinocytes themselves may release SP when they are stimulated by SP, and induce paracrine signaling via binding to the NK-1 receptor on keratinocytes, which ultimately induces cell proliferation as well as migration [8,30,31]. These and other studies showed the beneficial effect of SP on wound healing in vitro under normal conditions, and herein we show that SP is also effective in a serum-starved and hypoxia environment. Acute transient hypoxia or low oxygen is a primary mediator of the wound healing response via activation of hypoxia-inducible factor (HIF)-1, leading to the secretion of several cytokines/growth factors involved in several processes such vascularization, proliferation, and migration; however, chronic hypoxia leads to non-healing chronic open wounds [32,33]. Strategies to alleviate local hypoxia, such as by providing topical oxygen in a hyperbaric oxygen chamber, exist but are not readily available and have led to inconsistent results [34]. Therefore, topical treatments for chronic wounds must be able to work even under the suboptimal chronic wound environment. For this reason, we performed in vitro studies that mimic chronic wounds with low oxygen and low serum. We found that while low serum and hypoxia delayed wound healing in keratinocyte monolayers, exogenous SP was able to reverse these effects.

In our in vivo study, we used 15-week-old male and female mice because the wound healing process is known to slow down with increasing age, which may be due to a range of factors, including decreased neurogenic modulation, vascularity, and SP levels [35,36]. Therefore, we monitored the healing of full-thickness biopsy wounds on 15-week-old male and female mice. These animals are easy to maintain and cost-effective in comparison to SCI and diabetic animals. Most studies show that SP accelerates diabetic wound healing through modulating inflammatory responses, notably the activation of NF-κB and inflammatory cell density [8,11,37,38,39]. Another major mechanism of SP improving wound healing is angiogenesis acceleration [40,41,42]. SP may accelerate angiogenesis by modulating levels of transforming growth factor-β1 (TGF-β1), vascular endothelial growth factor (VEGF), tumor necrosis factor-α (TNF-α), and interleukin 10 (IL-10) during wound healing [13]. Additionally, SP has been reported to help repopulate stem cells, leading to enhanced wound healing [14,17]. SP also improves wound healing by promoting stromal maturation and re-epithelization [8,43,44]. These may be the mechanisms contributing to SP’s positive therapeutic effect on skin wound healing. Our study also showed that a single dose of exogenous SP enhanced the wound closure and improved the thickness of the dermis and epidermis in both male and female mice. This is supported by other studies including different parameters and different pathologies [3,8,22,36,45,46].

## 5. Conclusions

The data herein show that hypoxia and limited serum concentration significantly reduced HaCaT cell proliferation and migration and thereby slowed wound closure in vitro. Under these suboptimal conditions, exogenous neuropeptide SP significantly enhanced these functions of wound healing. Slow wound healing is a problem associated with aging, diabetes, and SCI. In full-thickness model wounds in 15-week-old male and female mice, a single exogenous application of SP enhanced wound closure in both genders and increased the thicknesses of epidermal and dermal layers of skin. Taken together, SP enhanced wound healing in vitro and in vivo in systems mimicking suboptimal chronic wound conditions.

## Data Availability

Data available on request.

## Supplementary Materials

The following are available online at https://www.mdpi.com/2227-9059/9/2/222/s1, Figure S1: Effect of different concetrations of SP on in vitro wound closure under normaxia (left column, N) or hypoxia (ritght column, H), and regular serum condition (upper row) or deficient serum conditions (lower row). Quantified wound healing images showing the fraction (%) of wound closure over 6 days in various culture environments (*n* = 6–15).
