# Supplementary material for: Neuropeptide Substance P Enhances Skin Wound Healing In Vitro and In Vivo under Hypoxia
Source: Biomedicines. 2021 Feb 22;9(2):222. doi: 10.3390/biomedicines9020222 (PMC7926396; doi:10.3390/biomedicines9020222)
Supplement: Supplementary file 1 [file biomedicines-09-00222-s001.pdf]

Supplementary Figure

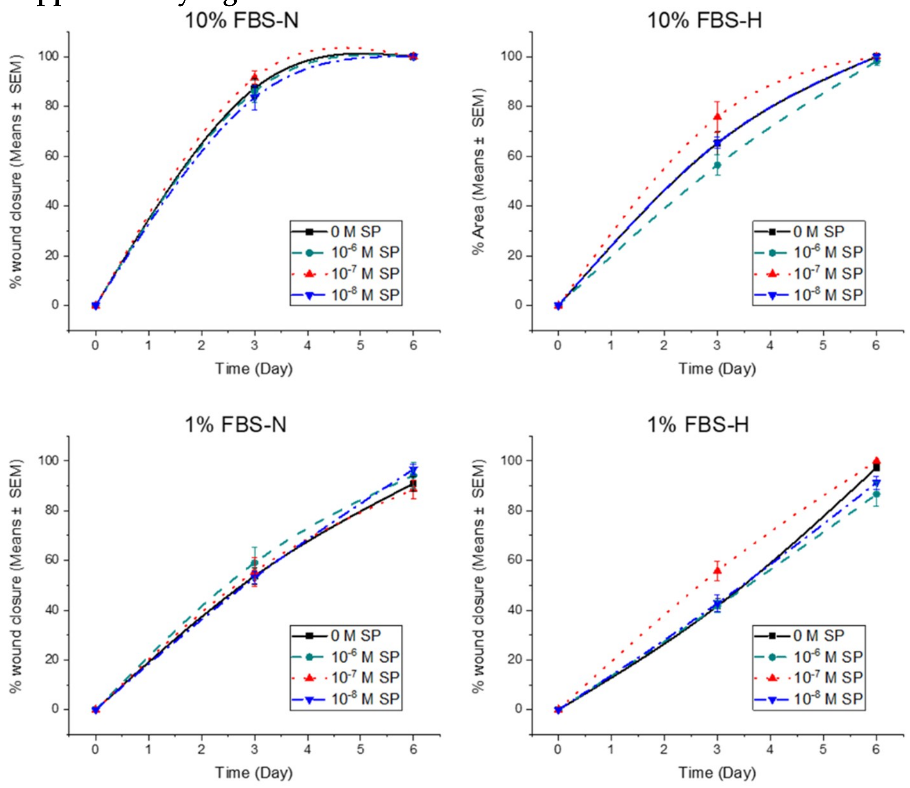

Figure S1. Effect of different concetrations of SP on in vitro wound closure under normaxia (left column, N) or hypoxia (ritght column, H), and regular serum condition (upper row) or deficient serum conditions (lower row). Quantified wound healing images showing the fraction (%) of wound closure over 6 days in various culture environments (n=6-15).
